# Exploration of the mechanism and therapy of ovarian aging by targeting cellular senescence

**DOI:** 10.1093/lifemedi/lnaf004

**Published:** 2025-01-23

**Authors:** Weicheng Tang, Kaichen Wang, Yourong Feng, Kuan-Hao Tsui, Keshav K Singh, Michael B Stout, Shixuan Wang, Meng Wu

**Affiliations:** Department of Obstetrics and Gynecology, Tongji Hospital, Tongji Medical College, Huazhong University of Science and Technology, Wuhan 430030, China; National Clinical Research Center for Obstetrical and Gynecological Diseases, Wuhan 430030, China; Key Laboratory of Cancer Invasion and Metastasis, Ministry of Education, Wuhan 430030, China; Tongji Medical College, Huazhong University of Science and Technology, Wuhan 430030, China; Department of Obstetrics and Gynecology, Tongji Hospital, Tongji Medical College, Huazhong University of Science and Technology, Wuhan 430030, China; National Clinical Research Center for Obstetrical and Gynecological Diseases, Wuhan 430030, China; Key Laboratory of Cancer Invasion and Metastasis, Ministry of Education, Wuhan 430030, China; Department of Obstetrics and Gynecology, Kaohsiung Veterans General Hospital, Kaohsiung 813779, Taiwan, China; Department of Obstetrics and Gynecology, Yang-Ming University, Taipei 112304, Taiwan, China; Department of Pharmacy and Graduate Institute of Pharmaceutical Technology, Tajen University, Pingtung 900391, Taiwan, China; Department of Genetics, School of Medicine, The University of Alabama at Birmingham, Birmingham, AL 35294, USA; Aging & Metabolism Research Program, Oklahoma Medical Research Foundation, Oklahoma City, OK 73104, USA; Oklahoma City Veterans Affairs Medical Center, Oklahoma City, OK 73104, USA; Department of Obstetrics and Gynecology, Tongji Hospital, Tongji Medical College, Huazhong University of Science and Technology, Wuhan 430030, China; National Clinical Research Center for Obstetrical and Gynecological Diseases, Wuhan 430030, China; Key Laboratory of Cancer Invasion and Metastasis, Ministry of Education, Wuhan 430030, China; Department of Obstetrics and Gynecology, Tongji Hospital, Tongji Medical College, Huazhong University of Science and Technology, Wuhan 430030, China; National Clinical Research Center for Obstetrical and Gynecological Diseases, Wuhan 430030, China; Key Laboratory of Cancer Invasion and Metastasis, Ministry of Education, Wuhan 430030, China

**Keywords:** ovarian aging, cellular senescence, oocyte, granulosa cells, senotherapy

## Abstract

The ovary is a crucial gonadal organ that supports female reproductive and endocrine functions. Ovarian aging can result in decreased fertility and dysfunction across multiple organs. Research has demonstrated that cellular senescence in various cell types within the ovary can trigger a decline in ovarian function through distinct stress responses, resulting in ovarian aging. This review explores how cellular senescence may contribute to ovarian aging and reproductive failure. Additionally, we discuss the factors that cause ovarian cellular senescence, including the accumulation of advanced glycation end products, oxidative stress, mitochondrial dysfunction, DNA damage, telomere shortening, and exposure to chemotherapy. Furthermore, we discuss senescence in six distinct cell types, including oocytes, granulosa cells, ovarian theca cells, immune cells, ovarian surface epithelium, and ovarian endothelial cells, inside the ovary and explore their contribution to the accelerated ovarian aging. Lastly, we describe potential senotherapeutics for the treatment of ovarian aging and offer novel strategies for ovarian longevity.

## Introduction

Ovarian aging refers to the progressive decline in ovarian function with age, characterized by reduced follicle numbers, decreased quality of oocytes, changes of menstrual cycle, decreased fertility, and ultimately menopause [1]. The decrease in estrogen levels due to ovarian aging can cause a series of clinical symptoms, such as vasomotor symptoms, osteoporosis, urogenital symptoms, neuropsychiatric dysfunction, cardiovascular diseases, endocrine diseases, and others [2]. This aligns with the previous perspective that ovarian aging acts as a sensor for the overall aging of the female body [3, 4]. In humans, ovarian function typically begins to decline around 35 years of age, progressively deteriorates after 37, and ultimately ceases reproductive function around age 50 [5]. Notably, a growing number of women have been opting to delay childbearing to later stages of life, often influenced by social factors. Consequently, the diminishing fertility attributed to ovarian aging poses a significant challenge in the field of reproductive medicine, as no treatment modality has been proven to delay ovarian aging.

Cellular senescence refers to an irreversible cell cycle arrest caused by multiple stress responses, including accumulation of advanced glycation end products, oxidative stress, mitochondrial dysfunction, DNA damage, telomere shortening, and chronic inflammation [6]. Cellular senescence exists throughout the life of multicellular organisms from development to death, and it also ubiquitously exists in both normal and senescent organs. Under physiological conditions, cellular senescence promotes organ differentiation and development by removing unwanted cells [7]. With the accumulation of time or the degree of aging, cellular senescence further promotes organ aging through a variety of pathways, such as reducing the number of cells, decreasing cell quality, reducing metabolic level, accumulating metabolic waste, producing reactive oxygen species (ROS), thus damaging the organ and weakening the physiological function of the organ. Recently, cellular senescence was hypothesized to contribute to the age-related decline in ovarian function [8]. Nevertheless, there remains a lack of a comprehensive theoretical framework concerning the role of cellular senescence in ovarian aging. Therefore, elucidating the role that cellular senescence may play in ovarian aging could lead to the development of novel therapies for reversing ovarian aging.

In this review, we will discuss various factors inducing ovarian cellular senescence, cellular senescence in six distinct cell types inside the ovary and discuss some pharmaceutical agents that specifically target cellular senescence.

## Various factors inducing ovarian cellular senescence

**Figure 1.**
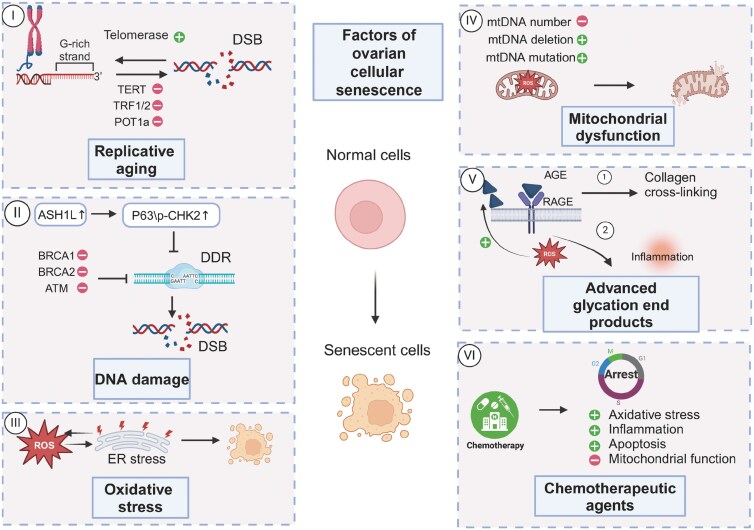
**Mechanisms of cellular senescence in the ovary.**Normal cells evolve into senescent cells through a series of mechanisms, including telomere shortening, DNA damage, the accumulation of oxidative stress, mitochondrial dysfunction, the presence of advanced glycation end products, and exposure to chemotherapy. Consequently, these mechanisms collectively contribute to the cellular transition towards senescence, highlighting the multifactorial nature of cellular senescence.

Ovarian aging is believed to result from several contributing factors, both intrinsic and extrinsic to the female body. Among these factors, cellular senescence is suggested to influence this process by possibly reducing follicle numbers, diminishing oocyte quality, and altering hormone secretion through different molecular pathways. Consequently, these processes contribute to the physiological decline of the ovary, ultimately resulting in ovarian aging. In this review, we will explain how ovarian cellular senescence is induced by the following factors: telomere shortening, DNA damage, accumulation of oxidative stress, mitochondrial dysfunction, chemotherapy, and advanced glycation end products (Fig. 1).

### Replicative ovarian cellular senescence

Replicative aging, commonly referred to as cellular senescence, is a phenomenon marked by the progressive shortening of telomeres. Telomeres, specialized DNA structures found at the ends of chromosomes, are essential for maintaining chromosome integrity. During each round of cellular division, telomeres undergo attrition due to the incomplete replication of terminal DNA sequences, ultimately imposing a limit on the replicative potential of somatic cells. Telomere shortening is associated with aging, and people with shorter mean telomere length than average have a higher risk of aging-associated diseases and mortality [9, 10]. Cell size and cycle increases correlate with telomere shortening, but not in cells overexpressing telomerase, suggesting telomerase prevents telomere-induced cellular senescence [11].

Research has shown the presence of telomerase in the normal human ovary, with its activity diminishing as individuals age. Moreover, human ovaries displaying follicle dysfunction show elevated telomerase activity, while those experiencing follicle depletion have notably reduced telomerase activity. Declining ovarian telomerase activity correlates with primordial follicle depletion during aging, suggesting its potential as an ovarian age marker [12]. In a groundbreaking 6-month randomized controlled trial involving 124 previously inactive adults, researchers have also suggested that activating telomerase could be a strategy to delay the ovarian cellular senescence [13]. Reduced expression of telomerase-associated proteins (TERT, TRF1, TRF2, and POT1a) during maternal aging correlates with telomere shortening in ovarian cells, potentially contributing to cellular senescence and age-related infertility [14, 15].

Telomeres play a crucial role in maintaining oocyte genomic integrity. Consequently, telomere shortening, which primarily results from elevated ROS, successive cellular divisions, and both genetic and epigenetic modifications, is intrinsically linked to oocyte aging [16]. Mice lacking telomerase (*Terc*^−/−^ mice) display a reduced number and function of oocytes with spindle defects and chromosomal misalignments *in vivo* [17]. Additionally, telomere signal intensity decline correlates with reduced expression of key telomere-maintenance proteins (TERT, TRF1, TRF2, and POT1a) in mouse follicles and oocytes. This suggests a potential link between telomere shortening in ovarian cells and reduced expression of these proteins [18]. In a case report of dyskeratosis congenita, reduced telomerase activity was associated with decreased fertility, characterized by oocyte senescence, reduced fertilization rates, an increased rate of aneuploidy in embryos, and shorter telomere lengths [19]. Scientists also conducted investigations on cumulus-oocyte complexes in an *in vivo* setting, highlighting that telomere length in cumulus cells can serve as an indicator for predicting oocyte quality and subsequent embryo development [20].

Telomere dysfunction and telomerase activity reduction have emerged as crucial factors in granulosa cell senescence in ovarian aging. Telomerase activity decreases in large follicles with atresia but remains stable in small follicles during mouse follicular development [15]. This decline in telomerase activity within granulosa cells can lead to its senescence, ultimately contributing to ovarian aging [21]. Furthermore, studies have found that patients with premature ovarian failure exhibit shorter telomere lengths and lower telomerase activity in granulosa cells and blood cells [22, 23], indicating the potential impact of telomere shortening in senescent granulosa cells on ovarian aging. Reduced ovarian telomerase activity affects telomere maintenance in granulosa cells during late follicular development in mice [24].

In conclusion, the process of telomere shortening and the reduction in telomerase activity have been implicated in ovarian cellular senescence, contributing to ovarian aging. Therefore, conducting comprehensive research on the factors that influence telomere and telomerase dynamics holds promise for generating novel insights and strategies aimed at preserving ovarian function and extending the period of ovarian youthfulness.

### DNA damage-induced ovarian cellular senescence

DNA damage refers to the destruction of structure and composition in cell DNA caused by endogenous or exogenous factors, one of which is DNA double-strand breaks. DNA damage activates a cellular defense mechanism known as the DNA damage response, which arrests cell cycle processes to allow repair mechanisms to correct the error [25]. When the cellular repair mechanisms become overwhelmed, senescence is induced through pathways such as the P53–P21/P16 pathways [26]. DNA damage response and DNA repair mechanism are essential for maintaining the metabolic balance of cellular senescence and aging phenotype [8]. Chemotherapy and aging increase DNA double-strand breaks in ovarian cells, while their repair maintains ovarian reserve and prevents cellular senescence [27, 28].

Genomic instability may lead to the onset of menopause. A genome-wide study of 201,323 women revealed menopause-related genes primarily cluster in DNA damage repair pathways, confirming DNA repair capacity’s role in ovarian aging [29]. Oocytes possess comprehensive DNA repair mechanisms that protect against senescence-inducing genetic damage from both internal and external sources. *Brca1/2* and *Atm* knockout mice showed that oocyte senescence results from impaired DNA repair, particularly through BRCA1/2 and ATM-mediated double-strand break repair, which naturally declines with age [30]. Ovarian ATM expression declines sharply after age 36, correlating with reduced fertility. *BRCA*-mutated women and mice show increased oocyte DNA double-strand breaks and decreased primordial follicles, leading to reduced ovarian reserve and earlier menopause. This demonstrates that impaired DNA repair accelerates ovarian aging. A recent study has identified a novel role of ASH1-like histone lysine methyltransferase (ASH1L) in the control of apoptosis during meiotic prophase I in mouse oocytes *in vitro* [31]. This study shows that overexpression of *Ash1l* gene can lead to premature up-regulation of P63 and phosphorylated CHK2 in the fetal ovary, which then causes defects in DNA double-strand breaks repair and induces cellular senescence, leading to ovarian aging.

A rhesus monkey study revealed that granulosa cells show age-related increases in DNA double-strand breaks and decreased repair capacity, suggesting these changes may drive cellular senescence and ovarian aging [32]. Moreover, TRDMT1, an RNA methyltransferase, enhances oxidative DNA damage repair, while *Trdmt1*-silenced mouse granulosa cells show increased susceptibility to H_2_O_2_-induced damage both *in vitro* and *in vivo* [33]. Therefore, they proposed that TRDMT1 can increase DNA damage repair in granulosa cells and that reduced TRDMT1 is associated with ovarian cellular senescence and decreased ovarian function.

In conclusion, DNA damage is a significant contributor to ovarian cellular senescence, with the failure of DNA repair mechanisms leading to the aging and apoptosis of oocytes and granulosa cells. This process results in a decline in ovarian function, ultimately accelerating ovarian aging. Therefore, in-depth research into the mechanisms of DNA repair is essential for the potential delay of ovarian aging.

### Oxidative stress-induced ovarian cellular senescence

Oxidative stress is a state of excess ROS production caused by loss of redox equilibrium. ROS, such as superoxide radicals, H_2_O_2_, hydroxyl radicals, and singlet oxygen, are free radical or non-free radical oxygenated molecules continuously generated by eukaryotic cells in metabolic processes as byproducts, which can lead to cellular senescence [34, 35]. Studies have indicated that oxidative stress is an important driver for ovarian aging, causing lipid peroxidation cascades which lead to oxidative damage, greatly affecting folliculogenesis, meiosis, and ovulation, thus causing ovarian aging [36, 37]. What’s more, ovary antioxidant content reduces with age, which represents a decreased ability to scavenge ROS [38].

Fenton reaction-induced oxidative stress from H_2_O_2_ disrupts meiotic spindle structure and chromosome alignment in mouse oocytes, impeding maturation and triggering senescence and apoptosis, thus contributing to age-related oocyte quality decline [39]. Additionally, ketoglutaric acid exhibits antioxidant effects and protects porcine oocyte meiosis through NRF2/ARE pathway activation, preventing senescence and apoptosis, suggesting antioxidant balance may delay ovarian aging [40]. Sequential injection of mare serum gonadotropin, hCG, and prostaglandin F2α in mice to induce repeated ovulation showed that increased lipid peroxidation and oxidative stress promote oocyte senescence [40]. GPX1 protein is a member of glutathione peroxidase family, which counteracts oxidative damage to protect cells [41]. In oocytes from aged monkey ovaries, GPX1 protein level was found to decrease [42]. What’s more, scientists showed that increasing *Gpx1* expression in oocytes can restore female mouse reproductive efficiency in antioxidant treatment [43, 44]. In conclusion, oxidative stress plays a pivotal role in detrimental effects on oocyte quality and oocyte senescence.

A study about the mechanism of cigarette smoke exposure on mouse diminished ovarian reserve found that redox imbalance in granulosa cells might be the reason for decreased ovarian follicle reserve of mice exposed to cigarette smoke [45]. Besides, FOXO1 transcription factor is a key regulator of oxidative stress. When FOXO1 is activated, it will cause senescence and apoptosis of granulosa cells, thus causing follicle atresia [46]. Studies also found that in patients with endometriosis, excessive ROS causes granulosa cells senescence by inducing endoplasmic reticulum (ER) stress, eventually contributing to endometriosis-associated infertility [47]. Excessive ROS damages ER redox homeostasis usually by inducing ER stress, which triggers unfolded protein response to restore ER homeostasis by activating genes encoding factors associated with protein folding and antioxidative ability [48]. Furthermore, there is a reciprocal interaction between persistent ER stress and heightened oxidative stress, resulting in a positive feedback loop [48]. A study about granulosa cells from *in vitro* fertilization patients found that antioxidant enzyme *SOD* gene expression level is lower in older women compared to younger women, which represents the decreased capacity of granulosa cells to protect the ovary against ROS with age [49]. What’s more, H_2_O_2_ neutralizing antioxidant enzyme *PRDX4* expression level in the ovarian tissues is also lower in peri-menopausal women than in young women [50]. It is believed by some researchers that the reduced ability of granulosa cells to counteract ROS is a reason for its senescence, and eventually leads to reduced follicle number, decreased follicle quality, and aberrant ovary reproductive endocrinology [51]. It is also found that the accumulation of ROS in the ovary decreases oocyte quality, induces granulosa cell apoptosis, and accelerates degeneration of the corpus luteum in bovine granulosa cells *in vitro* [52].

In conclusion, oxidative stress which is closely related to cellular senescence is involved in mediating the pathophysiological process of follicle development, ovulation, and aging of the ovarian. Maintaining the dynamic balance of free radical-antioxidant system is an important method to delay ovarian aging.

### Mitochondrial dysfunction-associated ovarian cellular senescence

Mitochondria, vital organelles in eukaryotic cells, are crucial for cellular energy supply, cell cycle regulation, and programmed cell death. Mitochondrial dysfunction is intrinsically linked to cellular senescence, playing a significant role in the mechanisms that induce and maintain the senescent phenotype [53]. Ovarian cells demand a high amount of energy to sustain their unique functions, including oogenesis and steroidogenesis, especially in oocytes and granulosa cells. Notably, oocytes are some of the most mitochondria-abundant cells in the body [54]. Consequently, mitochondrial dysfunction is intricately linked to ovarian cellular senescence. In addition to the natural aging of ovaries in all women, premature ovarian insufficiency (POI, also known as premature ovarian failure or premature menopause) is reported in about 2% of women worldwide [55, 56]. At least eight genes (*POLG1*, *TWNK*, *AARS2*, *CLPP*, *LRPPRC*, *MRPS22*, *LARS2*, *HARS2*) are reported to be involved in POI function all of which carry out important mitochondrial function [56]. Mutations in these genes impact multiple mitochondrial function including reduced mitochondrial DNA (mtDNA) content, mitochondrial transcription, mitochondrial translation, and mitochondrial proteostasis. Unfortunately, potential mitochondrial mechanisms leading to cellular senescence contributing to premature ovarian aging are not understood [57].

Mitochondria maintain their quantity, size, shape, and distribution through fission and fusion, also known as mitochondrial dynamics, thereby regulating mitochondrial function [58]. By comparing differences in the mitochondrial function of oocyte mitochondria in younger (under 35 years old) and older (over 40 years old) women undergoing *in-vitro* fertilization treatment, a study found that the quantity and quality of mitochondria significantly decrease during the aging process of oocytes [59]. Additionally, compared to younger women, the number of mitochondria in the granulosa cells of older women is significantly reduced [49]. In addition to the reduction in mitochondrial quantity, the imbalance of mitochondrial fusion and fission also leads to cellular senescence. MARCH5 protein regulates this balance, and its deficiency causes mitochondrial elongation, triggering cellular stress and senescence [60]. Moreover, the morphological changes in senescent granulosa cells are noticeable, such as elongation, swelling, and vacuolation [61]. Thus, disruption of mitochondrial dynamics is associated with cellular senescence. Accumulated dysfunctional mitochondria lead to increased oxidative damage, promoting the formation of megamitochondria and mitochondrial elongation. This further exacerbates mitochondrial dysfunction and induces cellular senescence [60, 62].

In senescent cells, the primary characteristics of mitochondrial dysfunction are the reduced respiratory capacity of each mitochondrion and the diminished mitochondrial membrane potential [58]. This leads to oxidative phosphorylation dysfunction, electron transport chain instability, and elevated ROS generation, thereby inducing ovarian cellular senescence [63]. Studies have found that excessive oxidative stress acts as a specific trigger for the senescence of oocytes, directly targeting the mitochondrial membrane. This disrupts oxidative phosphorylation within the mitochondria, resulting in decreased ATP production [46]. Additionally, an excess of ROS directly influences signaling pathways. For instance, by activating the MAPK–P53–SIAH1–TRF2 pathway, it induces telomere shortening and accelerates the senescence of human granulosa cells *in vitro* and *in vivo* [64]. Aged cow granulosa cells exhibit multiple mitochondrial dysfunction markers: decreased TOMM20 and TFAM, elevated ROS, disrupted membrane potential, and altered glucose metabolism with increased lactate production [65]. These findings strongly suggest that mitochondrial dysfunction plays a significant role in the senescence of oocyte and granulosa cells, potentially contributing to ovarian aging.

Apart from mitochondrial dynamics, oxidative stress, and the electron transport chain, mitochondrial dysfunction resulting from mitochondrial genome instability is also of paramount importance in ovarian cellular senescence. mtDNA dysfunction can manifest both quantitatively, for instance, through changes in mtDNA copy number and mtDNA deletions, and qualitatively, through strand breaks, point mutations, and oxidative damage [57]. Numerous studies have substantiated the relationship between mtDNA and reproductive capacity, indicating an inverse correlation between the mtDNA in oocytes and age, while demonstrating a positive correlation with ovarian reserve [57, 66]. Senescent oocyte and granulosa cells may be attributed to mitochondrial dysfunction resulting from inadequate mtDNA content and compromised mitochondrial biogenesis [67–69]. mtDNA compaction into dense nucleoids reduces copy number and transcription, causing mitochondrial protein dysfunction and cellular senescence. Ovarian cells’ weak antioxidant defenses make them particularly vulnerable to mtDNA stress damage [70]. The absence of histone protection and an efficient DNA repair mechanism in mtDNA results in its mutation rate being 25-fold higher than that of nuclear DNA [71]. An examination of 155 unfertilized MII oocytes from older women revealed the presence of a 4977 bp mtDNA deletion, which may be attributed to the homology between nuclear ATPase 8 and mitochondrial *MT_ND5* genes [72, 73]. mtDNA codes for essential electron transport chain and oxidative phosphorylation components. Its mutations impair energy production and increase oxidative stress, leading to cellular senescence [74].

**Figure 2.**
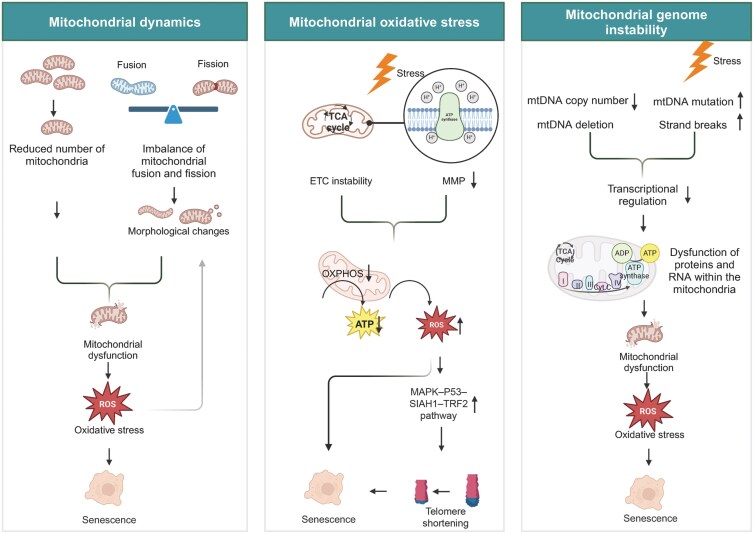
**Mechanisms of mitochondrial dysfunction in ovarian cellular senescence.**Mitochondria contribute to ovarian cellular senescence by modulating mitochondrial dynamics, mitochondrial oxidative stress, mitochondrial genome stability. Firstly, mitochondrial numbers decrease with age and an imbalance between mitochondrial fusion and fission results in abnormal mitochondrial morphology. All of the above can lead to mitochondrial dysfunction, resulting in the accumulation of oxidative stress and promoting cellular senescence. In addition, accumulated oxidative stress can affect mitochondrial morphology, which can further lead to mitochondrial dysfunction. Secondly, the instability of the mitochondrial electron transport chain coupled with the disruption of membrane potential can cause mitochondrial oxidative phosphorylation dysfunction. This, in turn, impairs ATP production and leads to an overproduction of ROS. On one side, the accumulation of ROS directly accelerates cellular aging; on the other side, it can induce telomere shortening through signaling pathways, thus promoting cellular aging. Thirdly, mitochondrial genome instability inhibits the regulation of mitochondrial transcription, thereby affecting the function of proteins in the mitochondrial electron transport chain. Consequently, this dysregulation of mitochondrial function results in oxidative stress, which subsequently leads to cellular senescence.

In conclusion, factors, such as mitochondrial dynamics, mitochondrial oxidative stress, and mitochondrial genome stability, all affect mitochondrial function and take part in cellular senescence leading to ovarian aging (Fig. 2). However, most of the research has predominantly focused on oocytes and granulosa cells, with a limited number of studies investigating other cell types in ovary. The role of mitochondrial dysfunction in other cells, such as ovarian theca cells and ovarian immune cells, in the context of ovarian aging remains an area ripe for further exploration.

### Accumulation of advanced glycation end products and ovarian cellular senescence

Advanced glycation end products (AGEs) are formed by glycation in or out of cells, which are a kind of metabolic production of nonenzymatic reactions between reducing sugars and free amines of proteins as well as of amino lipids and nucleic acids and increases progressively with age [75]. Furthermore, the accumulation of AGEs is a contributing factor to cellular senescence, and over time, the persistent buildup of AGEs can impair protein function and promote oxidative stress and inflammation [9, 76].

Research on human follicular fluid and AGEs has revealed that the buildup of AGEs within the follicle can diminish oocyte function potential by triggering an inflammatory response through the activation of ER stress within the follicular microenvironment [77]. In addition, AGEs accumulated in the microenvironment of oocytes of aging women can promote protein damage, oxidative stress, and inflammation, thereby promoting oocyte senescence. These effects are predominantly mediated by the receptor for AGEs (RAGE) [78]. RAGE is a kind of multi-ligand protein and has important pro-inflammatory effects [79]. Previous studies have demonstrated that AGEs and RAGE are expressed in ovarian granulosa cells, theca cells and luteal cells [80, 81]. The binding of AGEs to RAGE in ovarian cells can activate several inflammatory signaling pathways, including mitogen-activated protein kinase (MAPK), extracellular signal-regulated kinase 1/2 (ERK1/2), protein kinase C (PKC), and nuclear factor-kappa B (NF-κB) [82]. Activation of these pathways can contribute to the inflammatory state, cellular oxidative stress, DNA damage, and ultimately cellular senescence by upregulating ROS and senescence-associated secretory phenotype (SASP), such as tumor necrosis factor (TNF-α), interleukin-1 (IL-1), and interleukin-6 (IL-6) [83, 84]. Furthermore, research suggests that increased ROS results in an increased formation of AGE. The binding of AGEs to RAGE consequently upregulates the expression of RAGE itself, thereby perpetuating a vicious cycle [85].

There are two forms of soluble RAGE namely endogenous secretory RAGE (esRAGE) and soluble receptor for AGE (sRAGE), both of which can prevent excessive ligand binding to RAGE thus protecting cells from damage of AGE-caused inflammatory cascades [86]. A study examining the impact of age on AGEs and sRAGE levels in human follicular fluid and plasma indicates that older women show lower plasma sRAGE levels. This observation suggests that sRAGE secreted in older women is more used by its binding to AGEs, which are produced in excess during aging [87]. Notably, the researchers saw a higher concentration of sRAGE in the plasma of younger women (aged 35 years and below). Therefore, plasma sRAGE levels may indicate follicular microenvironment conditions. Follicular fluid sRAGE concentrations could serve as an oocyte quality marker to help select oocytes for improved fertilization outcomes [88].

It has been observed that AGEs can increase production of extracellular matrix (ECM) and that AGEs are associated with abnormal collagen cross-linking of human ovarian tissue [89]. AGEs stimulate lysyl oxidase activity in the ovary of rats *in vivo*, which plays an important role in collagen and elastin cross-linking of extracellular matrix organization [90]. ECM and cellular senescence interact reciprocally. Increased and cross-linked collagen, the primary ECM protein, can trigger cellular senescence. On the other hand, senescent cells produce SASPs, including metalloproteinases, promoting ECM accumulation, and enhancing senescence [91].

**Figure 3.**
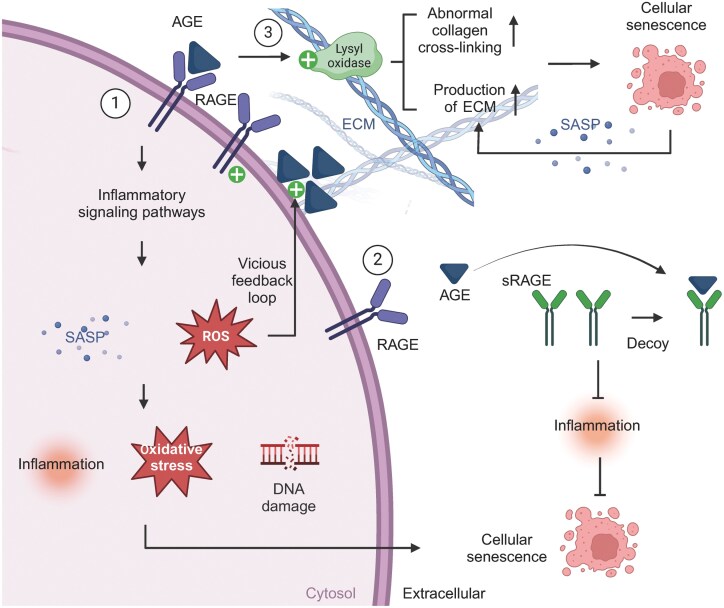
**Mechanisms of AGEs induced ovarian cellular senescence.**Firstly, AGEs, upon binding to its receptors (RAGE), triggers inflammatory signaling pathways, leading to the upregulation of ROS and SASPs. This cascade results in inflammation, oxidative stress and DNA damage, ultimately leading to cellular senescence. Additionally, ROS can enhance AGEs formation, and the interaction of AGEs with RAGE can further upregulate RAGE expression, perpetuating a vicious cycle. Secondly, another AGE receptor sRAGE, prevents excess ligand from binding to RAGE, thereby protecting cells from damage caused by the AGEs-induced inflammatory cascade. Thirdly, AGEs promote cellular senescence by upregulating abnormal collagen cross-linking and ECM formation in the ovaries. The SASPs produced by senescent cells also promotes ECM accumulation, thereby exacerbating cellular senescence.

The aforementioned studies collectively suggest that AGEs play a significant role in the regulation of the ovarian microenvironment. AGEs can directly induce protein damage, DNA damage, leading to oxidative stress and inflammatory responses (Fig. 3). The accumulation of AGEs during ovarian cellular senescence can consequently result in diminished ovarian function and fertility, thereby accelerating the process of ovarian aging.

### Chemotherapy-induced ovarian cellular senescence

**Figure 4.**
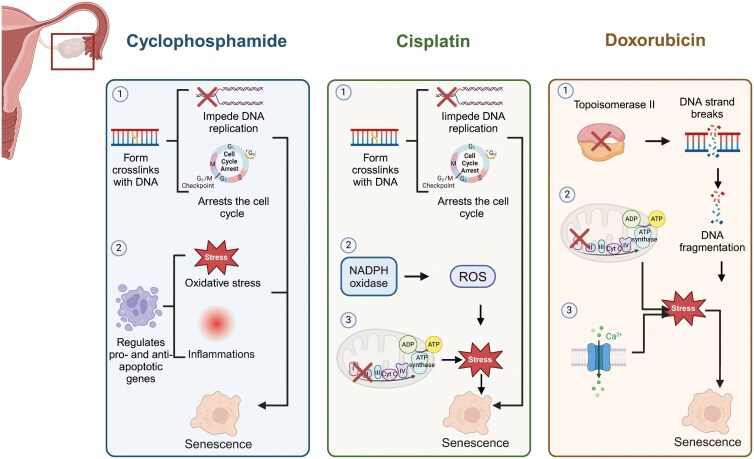
**Mechanisms of chemotherapy-induced ovarian cellular senescence.**CPM induces cellular aging through two primary mechanisms. On one hand, they form cross-links within DNA, thereby preventing DNA replication and arresting the cell cycle. On the other hand, these drugs regulate pro-apoptotic and anti-apoptotic genes, causing oxidative stress and inflammation, which further promotes cellular senescence. Besides forming cross-links within DNA, cisplatin facilitates ROS accumulation via the NADPH pathway and disrupt mitochondrial electron transport chain complexes, resulting in oxidative stress damage and then cellular senescence. Doxorubicin can inhibit topoisomerase II, leading to DNA breakage and fragmentation. The accumulated DNA fragments trigger cellular senescence. Additionally, these drugs can interfere with intracellular redox processes or disrupt intracellular Ca^2+^ homeostasis, affecting mitochondrial function and inducing oxidative stress, thereby promoting cellular senescence.

In addition to age-related ovarian aging, chemotherapy-induced ovarian aging represents another clinically significant manifestation of ovarian dysfunction. Chemotherapeutic drugs target both malignant and non-malignant cells, resulting in cellular senescence and apoptosis. Hence, senescence assumes a significant role in the *in vivo* response to chemotherapy [92]. In this section, we will elucidate the effects of several commonly prescribed chemotherapeutic drugs on ovarian cellular senescence (Fig. 4).

#### Cyclophosphamide

Cyclophosphamide (CPM), as a first-line chemotherapeutic agent, is extensively utilized in the treatment of various malignancies, including breast cancer, ovarian cancer, and pediatric hematologic tumors [93]. It serves as a model for chemotherapy-induced ovarian damage in both rodents and humans. CPM-damaged ovaries exhibit elevated follicle-stimulating hormone (FSH) and luteinizing hormone (LH), reduced estradiol, disrupted estrous cycles, depleted ovarian reserves, stromal fibrosis, and vascular damage [94]. By forming crosslinks with DNA, their metabolites impede DNA synthesis and function. Its active metabolite, phosphoramide mustard forms DNA adducts and double-strand breaks through covalent binding with DNA. These events impede DNA replication and arrest the cell cycle in the G2/M phase, resulting in cellular senescence [95]. CPM elevates the expression of cellular senescence markers, such as CDKN1A, CDKN2A, P53, and γH2AX, and concurrently reduces the numbers of primordial and primary follicles in mouse ovaries [96]. Furthermore, research has demonstrated that CPM induces ovarian apoptosis by regulating the expression levels of pro- and anti-apoptotic genes [97]. Cellular senescence and apoptosis on a large scale will lead to oxidative stress and inflammation in the ovary microenvironment, which explains why inhibiting oxidative stress and inflammation can alleviate damages caused by CPM [98]. CPM and its by-products are linked to the reduction of antioxidants, the creation of oxidative stress, and significant damage to mitochondria dysfunction [99]. Additionally, CPM has been shown to elevate pro-inflammatory cytokines such as IL-6, IL-8, and TNFα, concurrently with a decrease in the anti-inflammatory cytokine IL-10, thereby inducing an inflammatory response [100]. In summary, CPM induces cellular senescence in the ovaries through mechanisms including the facilitation of DNA damage, interruption of the cell cycle, augmentation of oxidative stress and inflammatory responses, and impairment of mitochondrial function.

#### Cisplatin

Moreover, as a first-line treatment for ovarian cancer, the gonadal toxicity of cisplatin in females warrants particular attention. Cisplatin often results in mild to moderate instances of amenorrhea and an elevated risk of ovarian failure and infertility [101]. Studies have suggested that cisplatin damages the ovary mainly by reducing ovarian reserve, decreasing female fertility, and increasing the number of atretic follicles [101]. The damage induced by cisplatin involves several key pathways, including DNA damage and oxidative stress, both of which are crucial processes in cellular senescence [102]. One of the fundamental mechanisms of cisplatin is its capacity to forge cross-links within DNA, an intricate process that disrupts DNA repair mechanisms and impedes cellular growth and division [101]. Moreover, by causing DNA damage, cisplatin activates cell cycle checkpoints, resulting in the arrest of the cell cycle and ultimately leading to cellular senescence [103]. Additionally, cisplatin contributes to cellular senescence through oxidative stress and the production of free radicals. Cisplatin facilitates the transformation of NADPH into NADP^+^, concurrently releasing electrons, thereby engendering an increased production of ROS [104]. The processes markedly intensify the oxidative stress though the disruption of the mitochondrial electron transport chain complexes and the depletion of antioxidants like superoxide dismutase and glutathione [105]. Concurrently, researchers have observed that following cisplatin intervention, the ovary of mice exhibit damaged mitochondrial cristae, which induce cellular senescence [106].

#### Doxorubicin

As a prominent anthracycline antibiotic, doxorubicin exhibits broad-spectrum antineoplastic activity, particularly in the treatment of hematological malignancies, whereas it also serves as a crucial therapeutic agent for solid tumors, including breast cancer, ovarian cancer, and thyroid carcinoma [107]. It has been reported that both adults and children undergoing doxorubicin treatment suffer from ovary damages, such as decreased ovarian reserve, amenorrhea, early menopause, low fertility, and even infertility [101, 108]. Studies have found that doxorubicin induces cellular senescence in ovarian cells, characterized by an elevated expression of senescence markers P16 and P21, and an increased positivity in SA-β-gal staining [109]. Doxorubicin also damages the ovary by causing irreversible DNA damage, increasing apoptosis, over-activating primordial follicle, and enhancing oxidative stress [110, 111]. Doxorubicin inhibits topoisomerase II, an enzyme crucial for DNA replication during G2/M phase by managing DNA strand breaks and resealing. By preventing topoisomerase II from resealing DNA nicks, doxorubicin causes DNA strand fragmentation, leading to cellular senescence and death [101, 112]. What’s more, doxorubicin triggers cellular senescence through increased ROS production and mitochondrial dysfunction. The redox reactions within the cell are mediated by the mitochondrial electron transport chain complexes, particularly Complex I, along with NADPH/cytochrome P450 and endothelial nitric oxide synthase. Within this electron transport chain, Complex I serves as the site of doxorubicin reduction. Consequently, doxorubicin can induce oxidative stress by disrupting the intracellular redox processes [113, 114]. Besides, doxorubicin can induce a disruption in the homeostasis of calcium ions (Ca^2+^), alter the transmembrane potential, and modify mitochondrial permeability, thereby precipitating cellular senescence through its influence on oxidative stress.

## Senescence of different ovarian cells and ovarian aging

**Figure 5.**
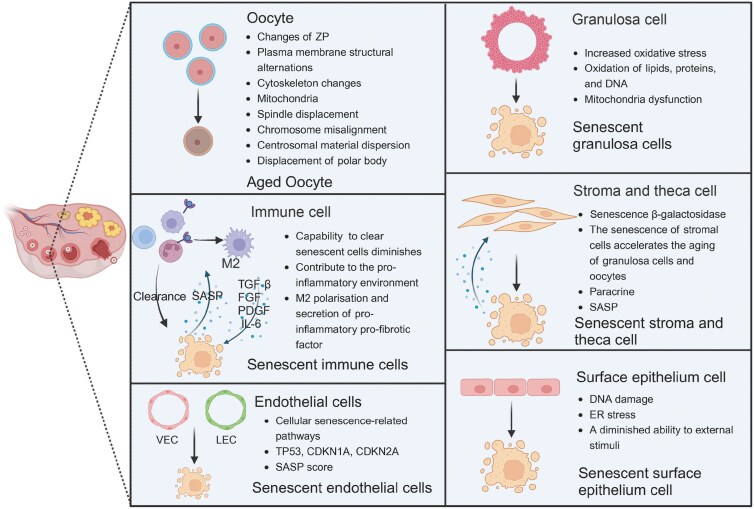
**The process of cellular senescence in various ovarian cells.**In the context of oocyte aging, some alterations within the zona pellucida, abnormalities in the plasma membrane structure and changes in the cytoskeletal architecture are observed. Granulosa cells enter a state of cellular senescence through processes such as increased oxidative stress and mitochondrial dysfunction. Stromal cells undergo cellular senescence via SASP, paracrine actions, and interactions with granulosa cells and oocytes. Following the senescence of immune cells, their capacity to eliminate aging cells diminishes, leading to a pernicious cycle through the further secretion of pro-inflammatory and pro-fibrotic factors. Epithelial cells experience cellular senescence as a consequence of DNA damage and endoplasmic reticulum stress. Endothelial cells, including vascular and lymphatic endothelial cells (VEC and LEC), were found to exhibit increased expression of cellular senescence pathways, SASP score, and senescence markers. ZP, zona pellucida.

The ovary mainly consists of oocytes, granulosa cells, theca, and stromal cells, endothelial cells, epithelial cells, immune cells, and smooth muscle cells [115, 116]. In ovarian aging, diverse types of ovarian cells will experience cellular senescence and they affect ovarian aging through different pathways. Next, we will discuss the senescence of diverse types of ovarian cells and how they accelerate ovarian aging (Fig. 5).

### Aging oocytes

It has been shown that age-related infertility can be ameliorated through oocyte donation from younger donors [117, 118]. Decreased oocyte function results in embryo developmental arrest, aneuploidy, and miscarriages. It is reported that the successful delivery rate per embryo is 43.2% in women under 35 years old and 15.1% in women aged 41–42 years, and it decreases to 5.9% in women over 42 years old [119]. Aneuploidy is the most common type of chromosomal abnormality related to oocyte aging, which is an abnormal number of chromosomes. The frequency of egg aneuploidies and trisomic pregnancies increases dramatically with maternal age, often leading to miscarriage. In addition, they explored the causes of increased chromosome missegregation in the oocytes of older women, primarily arising from meiosis I [120].

Ultrastructural changes in oocytes, including meiotic spindle disruption and zona pellucida sclerosis, significantly contribute to ovarian aging [121]. Research indicates that these changes encompass plasma membrane structural alterations, zona pellucida and cytoskeletal modifications, mitochondrial variations, spindle displacement, chromosome misalignment, centrosomal material dispersion, and polar body displacement [122]. A key marker of oocyte aging is the mtDNA copy number, which is significantly reduced in aged women or those with diminished ovarian reserve compared to younger women [123]. Additionally, aged oocytes often exhibit a higher risk of 4977-bp mtDNA deletion, impacting mitochondrial function and enzyme activities, including ATP synthases 6 and 8, cytochrome oxidase subunit 3, and NADH [73]. These oocytes also show decreased ATP production and downregulation of genes related to oxidative stress [124]. Furthermore, epigenetic changes, such as DNA, histone methylation, and acetylation, are involved in aged oocytes. Notably, altered acetylation at specific histone lysine residues and insufficient histone deacetylation can accelerate this process [125].

Overall, oocyte aging is a complex process with multiple contributing factors that affect female reproductive capability as women age. Notably, the aged oocyte mentioned above specifically refers to the decline in oocyte function with advancing age. Currently, the concept of oocyte senescence has been reported in only one study, whereas they experimentally detected SASP expression in primordial oocytes [126], so additional studies are needed to firmly establish if primordial follicles with senescent oocytes have already lost their capacity for folliculogenesis.

### Granulosa cell senescence

The follicle consists of primary oocytes surrounded by somatic granulosa cells. Granulosa cells play an important role in the maintenance of oocyte function, follicular development, ovulation, and hormone secretion [127]. Granulosa cells facilitate bidirectional communication with oocytes via paracrine factors and gap junction signals, thus enhancing oocyte growth and maturation. Therefore, senescence of granulosa cells could hamper their supportive role in oocyte development, leading to a decrease in oocyte quality and quantity, which culminates in a reduction of fertility associated with advancing age [128]. Conversely, compromised oocyte quality can also impair granulosa cell function and follicular development by endocrine, paracrine, and autocrine signals [129, 130]. Granulosa cells also play a crucial role in the growth and development of ovarian follicles. Their senescence often leads to arrested development or the emergence of dysfunctional follicles, which significantly contributes to decreased fertility [131]. Additionally, due to the hormonal secretion functions of granulosa cells, especially in the synthesis of estrogen, their senescence could result in hormonal dysregulation. Consequently, this can impact the menstrual cycle, potentially leading to the onset of menopauses [132].

It has been demonstrated that increased oxidative stress is an important contributor of granulosa cells senescence, characterized by an imbalance between the production of ROS and the antioxidant defense mechanisms [133]. Elevated levels of ROS lead to cellular damage through the oxidation of lipids, proteins, and DNA, contributing to cellular senescence. In addition, ROS can damage DNA, which is a typical feature of cellular senescence [134]. This oxidative damage triggers a cascade of molecular events, including the upregulation of senescence-associated markers such as p53, p16^INK4a^, and p21 in granulosa cells [135]. As a primary source and a target of ROS, mitochondria dysfunction also contributes to cellular senescence of granulosa cells, including a decrease in mtDNA copy number, alterations in mitochondrial ultrastructure, increase in mtDNA deletions and mutations, and altered mitochondrial membrane potential and metabolic function [127]. Senescent granulosa cells show a shift in energy metabolism, moving from a young phenotype that favors glycolysis and active production of pyruvate and NADPH, to an aged phenotype characterized by reduced aerobic glycolysis and mitochondrial respiration. This shift leads to a decrease in the support provided to oocytes, especially in terms of oxidative phosphorylation, which is crucial for oocyte maturation and overall reproductive success [136].

Overall, key factors, such as oxidative stress, DNA damage, and mitochondrial dysfunction, are instrumental in the senescence of granulosa cells. This process, in turn, profoundly affects follicular development, hormonal secretion, and the function of oocytes.

### Ovarian stroma and theca cell senescence

Stromal cells are a group of incompletely characterized cells, including interstitial cells and cells with the shape like fibroblast and spindle [137]. They form connective tissue that fills the space between the ovarian follicles and other structures within the ovary, supporting the formation of the follicular structure. Stromal cells also contain cells that can differentiate into various cell types, including theca cells [138]. Theca cells form a layer around the ovarian follicle and play a series of roles in follicle formation, interacting granulosa cells with oocytes and providing androgen required for follicle development and secretion of estrogen by granulosa cells [139]. The reduced production of steroids by theca-stroma cells is associated with decreased follicle number during ovarian aging [140].

Our team’s review on the ovarian stromal microenvironment and ovarian aging reveals that stroma and theca cells may experience age-related dysfunction through multiple regulatory pathways, including estrogen signaling, insulin pathways, and circadian rhythms. However, direct investigations into the aging of stroma and theca cells remain notably scarce [141]. Given their role as supportive cells in the ovary, the senescence of stromal cells has implications for the maintenance of ovarian structural integrity and the microenvironment. SA-β-gal is found in stroma-theca cells earlier than follicular cells, which indicates that senescence of stroma-theca cells may pace up ovarian aging [142]. Given the interactive role of stromal cells with granulosa cells and oocytes, the senescence of stromal cells also accelerates the aging of granulosa cells and oocytes. A study of bovine ovarian follicular development showed that ovarian theca cells can secrete factors that inhibit apoptosis to regulate cellular senescence and apoptosis of granulosa cells [143]. Studies have demonstrated that senescent ovarian theca cells damage follicles by producing C–C motif ligand 5 which can induce granulosa cell apoptosis, thus accelerating ovarian aging. Additionally, the SASP produced by senescent ovarian stroma and theca cells is involved in ovarian fibrosis, further influencing ovarian aging [144].

In summary, the senescence of ovarian stroma and theca cells has implications for ovarian structural integrity, the ovarian microenvironment, and the aging of granulosa cells and oocytes, ultimately contributing to ovarian aging.

### Immune cell senescence

Immune cells, such as T cells, B cells, dendritic cells, granulocytes, natural killer (NK) cells, natural killer T (NKT), and macrophages are identified in physiologically normal ovaries [145]. In spite of the small fraction of the immune cells in ovaries, they participate in many fertility-related processes in the ovaries, including follicle development, ovulation, and corpus luteum formation and regression [146, 147]. During ovarian aging, the quantity and composition of immune cells undergo significant transformation, accompanied by a concurrent alteration in their functional capabilities. A study utilizing single-cell transcriptome sequencing to explore the murine ovarian immune system uncovered that, there is a notable decrease in the proportion of most innate immune cells (macrophages, natural killer cells, ILC1, and NKT cells), shifting toward adaptive immunity (evidenced by an increased proportion of CD3^+^ lymphocytes) with advancing age [145]. The decrease of macrophages could lead to reduced fertility due to impairment of the ovarian blood vessels [148]. Additionally, the diminished capacity to immune response of macrophages and dendritic cells has been shown to result in a decreased number of ovulated oocytes, increased follicular atresia, and delayed progression of the estrus cycle [148, 149]. Also, Ma et al. found that naive CD4^+^ T cells in aged mice (21–22 months) are significantly less than those in young mice, suggesting that the decrease of CD4^+^ T cells may represent a decline in immune competence, thus leading to age-related decline in fertility [150]. Another single-cell transcriptomic study on ovarian aging in mice revealed a significant increase in immune cells within aged ovaries, with the proportion of lymphocytes showing the most substantial rise [151]. Moreover, a pronounced accumulation of CD3^+^ lymphocytes within the ovarian milieu has been correlated with a diminished follicular reserve [152]. During ovarian aging, the percentage of total B cells is higher in 12-month-old mice compared to 2-month-old mice. The infiltration of B cells in ovarian aging may contribute to the pro-inflammatory environment.

Senescent cells contribute to an inflammatory environment through the SASP, leading to immune cell response and clearance via phagocytosis (by macrophages) or killing (by natural T (NT) cells or NK cells) [153]. Granulocytes exhibited elevated expression of CCR1, a receptor for several SASP chemokines, while old NKT cells had higher levels of CD74, a receptor for MIF, another SASP member [154]. They all engage in the clearance of cells secreting the SASP. As age advances, the capability of immune cells to clear senescent cells diminishes, so resulting in an intensified extent of cellular senescence. In addition, it has been reported that excessive accumulation of lipofuscin, a biomarker of senescent cells, is associated with the status of macrophages during ovarian aging [155]. There is a shift in macrophages towards the M2 phenotype with aging, which promotes ovarian fibrosis through the secretion of TGF-β, FGF, PDGF, and IL-6 [47]. After using the anti-fibrosis drug BGP-15 to target the M2 subpopulation, there was a reversal in ovarian fibrosis, leading to improved ovarian function and better fertility in females [156].

Immune cells in ovaries play crucial roles in fertility-related processes and undergo significant changes in quantity, composition, and function during ovarian aging, leading to altered immune responses and fertility decline.

### Ovarian surface epithelium senescence

The ovary surface epithelium (OSE) covers the ovary and is composed of flat epithelial cells and cuboidal epithelial cells, which have fewer distinguishing features compared to other cells [157]. OSE is involved in material transportation to and from the peritoneal cavity and cyclically regulates ovulatory ruptures and repair [158]. OSE arouses the concern of scientists since it is considered as the major source of ovarian cancer [159]. Also, cellular senescence in OSE can affect the process of ovarian aging.

An important factor for OSE senescence is ovulation, which breaks surface epithelial lining integrity and induces OSE cell stress by physical force and changes in paracrine signaling [160]. A study has shown that the rate of OSE invagination and layering increases with ovulation number, which provides evidence for the influence of ovulation on OSE senescence [161]. During the process of ovulation, the DNA of OSE cells at the ovarian rupture site is observed to be damaged, which is another indicator of OSE senescence [162]. ER stress also plays an important role in OSE senescence by inducing unfolded protein response, thus accelerating the senescence of OSE cells [163]. In ovaries after menopause, OSE height will decrease and the epithelial layer is prone to develop invaginations and inclusion cysts, and such alternation has the risk of deteriorating into epithelial ovarian cancer [164]. Simultaneously, the senescence of OSE results in a diminished ability of the ovary to withstand external stimuli, leading to ovarian damage [165]. In all, the OSE, involved in material transport and ovulatory repair, is a key site for ovarian aging and cancer, with senescence influenced by factors like ovulation and ER stress.

### Ovarian endothelial cells senescence

The ovary is endowed with a rich vascular system, encompassing both blood vessels and lymphatic vessels. Notably, endothelial cells constitute a critical component of both these vascular and lymphatic systems [166, 167]. A study that mapped the single-cell atlas of ovarian aging in mice identified that ovarian endothelial cells consist of both vascular and lymphatic endothelial cells. Vascular endothelial cells, in particular, demonstrated pronounced age-associated changes, such as DNA damage and disruptions in cell cycle regulation. Correspondingly, cellular senescence markers such as TP53, CDKN1A, and CDKN2A significantly increased with the senescence of vascular endothelial cells [151]. Moreover, our analysis of the single-cell atlas of human ovarian aging demonstrated a significant upregulation of cellular senescence-related pathways within ovarian endothelial cells throughout the aging process [168]. This was coupled with a notable rise in SASP score levels, and the expression of the key senescence marker CDKN1A was markedly increased in these cells. These findings further substantiate the presence of senescence in ovarian endothelial cells during ovarian aging. However, the senescence of ovarian endothelial cells remains a newly discovered phenomenon, necessitating further research to explore its underlying mechanisms in ovarian aging.

Given the diverse ovarian cellular senescence types, therapeutic strategies have evolved to specifically target these senescent cell populations. Senolytics are specifically designed to induce apoptosis in senescent cells. These cells, characterized by their non-dividing state, are notorious for secreting a variety of detrimental substances that contribute to inflammation and tissue damage. The principal objective of senolytics is to purge these harmful cells from the body, thereby mitigating the adverse effects associated with aging and fostering a healthier aging process [169]. Senomorphic agents, distinct from those that induce cell death, strategically inhibit senescence initiation. Their primary goal is to suppress the harmful aspects of these cells, especially the release of harmful compounds, collectively termed the SASP. By regulating both the behavior and secretions of senescent cells, senomorphics are designed to diminish the adverse effects these cells exerting on the body, thus offering a nuanced approach to cellular aging without necessitating cell removal [169]. This classification, though widely accepted, is not rigid. Intriguingly, a drug’s role may shift from senomorphic in one cellular context to senolytic in another, reflecting its versatile nature. Detailed strategies targeting ovarian cellular senescence will be described later in this review.

## Cellular senescence-targeted drugs for ovarian aging treatment

In recent years, the field of senotherapy has made significant strides in addressing the challenges posed by cellular senescence like idiopathic pulmonary fibrosis, atherosclerosis, and osteoarthritis [8]. Herein, we explore novel therapeutic strategies aiming to mitigate the detrimental effects of senescent cells on the ovary (Table 1).

**Table 1.** Agents and their influence on ovarian cellular senescence.

Compound	Targets	Alterations in cellular senescence	Activity in ovarian function	Species	Reference	Development status
Dasatinib and quercetin	Pan tyrosine kinases/numerous	Decreased expression of P16, P21, and lipofuscin staining	Diminished the presence of senescent cells	Mice *in vivo*	[170]	Effect of dasatinib and quercetin in aging (NCT04946383) Effect of dasatinib and quercetin in Alzheimer’s disease (NCT05422885)
		Suppress the secretion of IL-1β, IL-6, P16, and P21	Improve oocyte quality and increase the ovarian reserve	Mice *in vivo*	[171]	
		Reduce SA-β-gal staining positive areas and levels of IL-6, P16, and P21	Not notably evident	Mice *in vivo*	[109]	
Rapamycin	mTOR	Reduce ovarian IL-1A, IL6, and TNF-α Improve mitochondrial function	Prolong ovarian lifespan, increase oocyte quality, and improve ovarian microenvironment	Mice *in vivo*	[172]	Effect of rapamycin in ovarian aging (NCT05836025) Effect of rapamycin in women with diminished ovarian reverse (ChiCTR2300069828) Effect of metformin in longevity (NCT04488601, NCT02874924, NCT05836025, NCT04994561)
		Reduce DNA damage	Inhibit apoptosis of developing follicles	Mice *in vivo*	[173]	
		Reduce cell apoptosis	Prevent the primordial follicle activation	Mice *in vivo*	[174]	
Metformin	IKK and/or NF-κB	Reduced senescence marker P16 and oxidized metabolites	Increased ovarian endocrine function and healthy follicles	Mice *in vivo*	[175]	Effect of metformin in ovarian toxicity during chemotherapy for early breast cancer [176] (ChiCTR1900023487) Effect of metformin in longevity (NCT02432287, NCT03309007, NCT01765946, NCT04994561)
		Reduce IL-1β, IL-6, P16, P21 and SA-β-gal activity	Improve oocyte quality and increased the ovarian reserve	Mice *in vivo*	[171]	
		Altering the proportion of immune cells	Prevented age-associated ovarian fibrosis	Mice *in vivo*	[177]	
Resveratrol	SIRT1	Improve mitochondrial function	Improve oocyte quality	Mice *in vivo*	[178]	Effects of resveratrol in oocyte quality of women undergoing *in vitro* fertilization (NCT06235294) Effect of metformin in longevity (NCT01126229, NCT02123121, NCT04994561)
		Suppress oxidative stress	Improve ovarian function	Rats *in vivo*	[179]	
		Alleviate inflammation, ER stress and IL-1β, TNF-α, IL-8	Improve oocyte and reduce atretic follicles	Fish *in vivo*	[180]	
Melatonin	Numerous	Oxidative stress, autophagy, ER stress, advanced glycation end-products, mitochondrial dysfunction and telomeres	Regulate ovarian function and enhance follicle growth	Hens and mice *in vivo*	[181–183]	Effect of melatonin on clinical outcome of women undergoing *in vitro* fertilization (ChiCTR2100045552, ChiCTR-INC-16009306) Effect of metformin in anti-aging (NCT03954899, NCT05440734, NCT03490825)
Coenzyme Q10	Mitochondrial respiratory chain	DNA damage and apoptosis Mitochondrial respiratory activity and glucose uptake	Improve ovarian quality Enhance fertility	Mice and pigs *in vitro* Mice *in vivo*	[184]	Effect of coenzyme Q10 in fertility in elderly patients (NCT02010164) Effect of coenzyme Q10 in aging (NCT02012322, NCT03893864)
		Mitochondrial function	Improve pregnancy outcomes	Human *in vivo*	[185, 186]	

### Dasatinib and quercetin

Dasatinib is an FDA-approved small-molecule multikinase inhibitor that effectively inhibits kinases of BCR-ABL family and SRC family [187]. Quercetin is a flavonoid with antihypertensive, anti-hyperlipidemic, anti-hyperglycemia, antioxidant, antiviral, anticancer, anti-inflammatory, anti-microbial, neuro-protective, and cardio-protective effects [188]. Combined used, dasatinib and quercetin (D + Q) induce apoptosis in senescent cells. They have been reported to have anti-aging effects (at concentrations of dasatinib: 5 mg/kg and quercetin: 10–50 mg/kg), increasing the average lifespan of mice and alleviating many age-related diseases, such as neurodegeneration, chronic kidney disease, and intervertebral disc degeneration, etc [189, 190]. The same effect is also found in initial trial in human (at concentrations of dasatinib: 100 mg/day and quercetin: 1000–1250 mg/day) [191, 192]. Research has shown that D + Q can alleviate inflammation in adipose tissue in the elderly. In aged mice (18–22 months), D + Q treatment (dasatinib: 5 mg/kg and quercetin: 50 mg/kg, administered monthly for 2 months) reduces the senescence markers like SA-β-gal, *Cdkn1a*, *Cdkn2a* gene in adipose tissue. Additionally, D + Q contributes to the decrease in several SASP-related factors, notably MCP1, TNF‐α, IL‐1α, IL‐1β, and so on [193]. D + Q (dasatinib: 5 mg/kg and quercetin: 50 mg/kg) can also reduce the degree of uterine fibrosis by reducing senescence markers (CDKN1A, CDKN2A), SASP factors, and the concentration of ROS in aged female C57BL/6 mice (18–20 months) [194].

The application of D + Q in safeguarding ovarian function has shown promising results. A 4-month regimen of D + Q (dasatinib: 5 mg/kg and quercetin: 50 mg/kg) markedly diminished the presence of senescent cells in ovaries. This effect is primarily due to the decreased expression of senescent cell markers, including CDKN1A, CDKN2A, and lipofuscin staining [170]. As previously discussed, addressing ovarian damage induced by cisplatin requires effective treatment strategies. In an ovarian aging mice model, mice were pre-treated with dasatinib (5 mg/kg) and quercetin (50 mg/kg) two times weekly for 4 weeks, followed by intraperitoneal cisplatin injections (2.5 mg/kg, three times per week for 3 weeks) after 1 week of pre-treatment. This intervention has been shown to improve oocyte quality and increased the ovarian reserve [171]. This is facilitated through the suppression of SASP markers and pro-inflammatory factor secretion, such as IL-6 and IL-1β. Another study discovered that D + Q (dasatinib, 5 mg/kg; quercetin, 50 mg/kg) administered by oral gavage every other day for 3 weeks significantly mitigates the increase in senescent cells within the ovaries induced by doxorubicin. This is shown by a reduction in SA-β-gal staining positive areas and lower levels of SASP markers (CDKN1A, CDKN2A, IL6, and MCP1). Despite these cellular changes, the improvement in overall ovarian function was not notably evident [109].

Hence, dasatinib and quercetin may be used to remove senescent cells from the ovaries. However, there is a slight concern regarding the use of dasatinib, as our study found that there was a risk of increased β-gal positivity and *CDKN2A* expression in granulosa cells when dasatinib was used alone at a concentration of 100 nM [195]. Further research is necessary to delve into the relationship between D + Q and cellular senescence within the ovaries.

### Rapamycin

Rapamycin (clinically known as sirolimus, an anti-fungal agent) has been shown to prolong the lifespan of mice, yeast, worms, and flies. It can also prevent age-related diseases in rodents, dogs, primates including humans [196]. Many studies have found that rapamycin effectively suppresses SASP markers and then inhibits cellular senescence whereas it is now known as one of the top senomorphics that help to mitigate the SASPs secreted by senescent cells, without inducing cell death [197, 198]. Its senomorphic properties and role in extending lifespan are predominantly attributed to its capacity to inhibit mTOR signaling, which is achieved primarily through the diminution of phosphorylation of S6K and 4E-BP, which are downstream effectors of TORC1 [199]. The alternative mechanisms through which rapamycin might regulate senescence include the activation of the NRF2 pathway and the attenuation of NF-κB activity, a process which consequently diminishes the production of IL-1α [200].

Rapamycin (4–5 mg/kg body weight every other day for 24–93 days) has been reported to extend ovarian longevity and inhibit cellular senescence of ovaries [174, 201]. Short-term administration of rapamycin (2 mg/kg daily intraperitoneal injection for 2 weeks) can prolong ovarian lifespan, increase oocyte quality, and improve ovarian microenvironment through the mTORC1 signaling pathway in 8-week-old and 8-month-old mice [172]. After rapamycin treatment, a notable diminution in ovarian SASP factors (IL‐1α, IL6, and TNF-α) has been observed. Additionally, rapamycin contributes to the amelioration of oocyte quality through alterations in mitochondrial membrane potential and its functions. In addition to natural aging, rapamycin protects against chemotherapy-induced ovarian aging. Researchers suggest that rapamycin (5 mg/kg intraperitoneally injected once daily for 30 days) can inhibit apoptosis of developing follicles by reducing DNA damage in 6-week-old mice [173]. They also show that administration of rapamycin (5 mg/kg) for 30 days demonstrated a favorable safety profile in mice, with no significant alterations in serum biochemistry (ALT, AST, CR), organ histopathology, or long-term body weight trajectory, despite transient weight loss during treatment.

The recurring finding in numerous studies that rapamycin modulates cellular senescence and maintains ovarian function highlights its significance in fertility preservation.

### Metformin

Metformin, originally developed for type 2 diabetes, has expanded to treat a range of age-related conditions, including insulin resistance, liver and heart diseases, cancer, and neurodegenerative disorders [202]. In addition, metformin has also been demonstrated to delay aging and prolong lifespan in various animals, such as worms, drosophila, and rodents [203, 204]. Extensive research confirms that metformin effectively reduces cellular senescence and SASPs. Specifically, metformin has been shown to decrease cellular SA-β-gal activity and lower the expression of SASP [202, 205].

Qin et al. found that metformin-treated mice (28-week-old mice were fed on chows supplied with 100 mg/kg metformin for 6 months) showed increased levels of serum E2 hormone, a higher percentage of regular estrous cycles, increased primordial and primary follicles and healthy follicles, along with reduced levels of senescence-associated protein P16 and oxidized metabolites [175]. The researchers hypothesized that metformin could delay mouse ovarian aging probably by inducing the expression of SIRT1, a protein related to ovarian aging, and reducing oxidative damage. Besides, a prospective, randomized, double-blind, placebo-controlled study including 314 women with breast cancer found that metformin attenuated cisplatin-induced ovarian damage. These women took oral metformin as prescribed, 500 mg daily for the first week of chemotherapy, 1000 mg daily for the second week, and 2000 mg daily for the third week to 1 month after the end of chemotherapy [176]. This process occurs through the reduction of SASP and elevated SA-β-gal activity caused by cisplatin [171]. What’s more, it has been demonstrated that metformin can prevent age-associated ovarian fibrosis in the human ovary after menopause by altering the proportion of immune cells, which plays a role in recognizing and clearing senescent cells [177]. Further research is imperative to delve into the intricate relationship between metformin and the preservation of ovarian function.

### Resveratrol

Resveratrol, a SIRT1 activator, is a natural phenolic compound with various bioactivities, such as antioxidant, anti-inflammatory, immunoregulatory, antihypertensive, and hypolipidemic effects. It can also prevent and manage cancer, cardiovascular diseases, neurodegenerative diseases, and obesity [206]. Recently, many studies have empathized its potential in treating aging and age-related diseases [207]. In neural cells, resveratrol has been seen to alleviate senescence by engaging SIRT1-mediated STAT3 signaling pathways. Additionally, resveratrol demonstrates its senescence-inhibitory prowess in human bone marrow stromal stem cells and exhibits an ability to suppress SASP factors in vascular smooth muscle cells. Thus, resveratrol functions as a senomorphic agent, efficaciously forestalling cellular senescence and curtailing SASP expression [208, 209].

It is found that 25-week-old mice fed with 6 g diet per day containing 0.04% resveratrol for 22 weeks can improve oocyte quality and is beneficial to female fertility, which may attribute to the increase of mitochondrial membrane potential and ATP content [178]. Besides, resveratrol intraperitoneal injection 20 mg/kg/day for 45 days can improve ovarian function of aged mice by suppressing oxidative stress in 15-month-old rats [179]. Also, resveratrol is reported to delay ovarian aging in fish by alleviating inflammation, ER stress, and cellular senescence [180]. It reduced the SA-β-gal activity and SASP, such as IL1-β, IL-8, and TNF-α. What’s more, Wu et al. found that resveratrol (30 mg/kg/day administrated by gastrogavage on alternating days for 2 weeks) can protect oogonial stem cells from apoptosis and treat ovarian aging in 6-week-old mouse ovary damaged by chemotherapy [210]. In summary, resveratrol can reduce the production of SASP, ROS, and AGE, decrease DNA damage, extend telomere length, and increase telomerase activity, thus delaying cellular senescence and treating ovarian aging.

### Melatonin

Melatonin is a pineal hormone associated with circadian rhythm, which can also be synthesized in the heart, liver, placenta, skin, kidney, and gut [211, 212]. In addition, melatonin regulates the levels of human gonadotropin and steroid hormones and affects the onset of puberty, sexual maturation, folliculogenesis, ovulation, pregnancy, and menopause [213]. It can also delay cellular senescence by reducing ROS generation, lightening inflammatory response, and increasing telomere length [214]. When exerting antioxidant effects, melatonin, along with its metabolites which act as a kind of antioxidant, form an antioxidant cascade to produce radical scavenger products, limiting oxidative damage through various mechanisms [215]. Apart from reducing ROS concentrations, melatonin can also interact with non-radical oxidants such as hydrogen peroxide, singlet oxygen, and peroxynitrite [216]. Melatonin can mitigate inflammatory response. In rodent studies, added exogenous melatonin decreased the level of pro-inflammatory cytokines, IL-1 β and TNF-α, and increased the level of anti-inflammatory cytokines IL-4 in serum [217].

It has been demonstrated that melatonin has great potential in ovarian aging treatment, mainly through oxidative stress, autophagy, ER stress, advanced glycation end-products, mitochondrial dysfunction, and telomeres [218]. Melatonin injected with 1 and 15 mg/kg can delay mouse ovarian aging by slowing down the decrease of ovarian reserve [181]. In addition, the study evaluated the safety of melatonin. They found that long-term intake of 15 mg/kg melatonin did not affect the body development and visceral indices of the mice, and did not affect the health status of the mice, such as body temperature and blood biochemical indices. It is found that melatonin (10-week-old mice were given melatonin-containing water 100 μg/mL until 43 weeks of age) can delay ovarian aging by various pathways such as alleviating oxidative damage, maintaining telomere length, inducing SIRT expression, maintaining ribosome function and reducing autophagy [182]. Besides, melatonin administered intraperitoneally at a dose of 20 mg/kg for 28 days can regulate ovarian function and enhance follicle growth in aging hens [183]. What’s more, *in vivo* studies also revealed the effect of oral melatonin (3 mg/day for 2 weeks) on promoting fertilization rate as well as embryo development in assisted reproductive technology [219].

### Coenzyme Q10

Coenzyme Q10 (CoQ10) is integral to ATP synthesis within the mitochondrial respiratory chain. Its reduced form, ubiquinol, protects biological membranes from lipid peroxidation by regenerating vitamin E. Research indicates that adding 50 μM CoQ10 to culture media can reverse age-related declines in oocyte quality by suppressing DNA damage and apoptosis in mouse and pig models [184]. Injection of 0.084 mg/kg/week CoQ10 for 12 weeks into 9-month-old mice has shown increased mitochondrial respiratory activity and glucose uptake in cumulus cells, thereby enhancing fertility [220]. Human studies reveal that plasma CoQ10 levels decrease with age, and supplementation with CoQ10 may improve pregnancy outcomes in infertility patients by enhancing mitochondrial function [221]. Aging leads to a reduction in the expression of CoQ10 synthesis genes in human cumulus cells, suggesting that CoQ10 supplementation could mitigate the effects of aging. CoQ10 levels in follicular fluid correlate with embryo quality and pregnancy rates, indicating its benefits extend to both oocytes and cumulus cells [222].

Further studies demonstrate that supplementing CoQ10 (oral administration of CoQ10 600 mg for 60 days) improves ovarian responsiveness in young women with diminished ovarian reserve, enhancing fertilization rates and increasing the number of high-quality embryos, though it does not significantly affect clinical pregnancy rates, miscarriage rates, or live birth rates [185]. In older women, 50 μM CoQ10 supplementation significantly improves oocyte maturity during *in vitro* maturation and reduces aneuploidy rates post-meiosis. However, similar effects are not observed in younger women. These findings underscore the potential of CoQ10 supplementation as a therapeutic strategy for improving reproductive outcomes, particularly in older women [186].

## Conclusions and perspectives

With the aging of the body, senescent cells in tissues and organs will gradually accumulate, thus causing aging and dysfunction of organs and tissues. Ovarian aging, serving as the pacemaker of female aging, is likely propelled by cellular senescence. Telomere shortening, oxidative stress, DNA damage, mitochondrial dysfunction, chemotherapy, and advanced glycation end products are the primary factors contributing to the senescence of ovarian cells. However, research on ovarian cellular senescence is still in its preliminary stages. The hierarchy and temporal sequence of cellular senescence in different ovarian cell types remains to be elucidated—specifically, whether senescence in one cell type triggers a cascade of senescence in others, and which cell type’s senescence plays the predominant role in ovarian aging. The molecular mechanisms regulating ovarian cellular senescence require further investigation. Additionally, current anti-senescence interventions shown to be effective for ovarian aging generally have broad effects on cellular senescence across multiple tissues. There is still a lack of targeted therapeutic approaches specific to ovarian cellular senescence. Thus, there is still a long way to go before they can be applied clinically.

Given the complex etiology and pathogenesis of ovarian aging, accurately evaluating ovarian reserve has become increasingly important for both clinical management and research purposes. Current clinical assessment of ovarian aging primarily relies on hormonal measurements (such as FSH and AMH levels), ultrasound examination (antral follicle count), and evaluation of clinical symptoms. With the advancement of technology, several novel approaches have emerged for more sensitive and comprehensive evaluation of ovarian aging. Recent studies have demonstrated that serum levels of cell-free mtDNA, microRNA, and metabolomic profiles could serve as promising biomarkers for ovarian aging [223–225]. In addition, comprehensive prediction models based on machine learning have been developed and applied in clinical settings to forecast ovarian aging [226–228]. For example, a recent study by our research team has demonstrated the utility of integrating multiple parameters, including hormonal markers, ultrasound features, and clinical factors, to quantify ovarian reserve with high accuracy [229]. Future research may focus on validating the diagnostic accuracy, cost-effectiveness, and practical applicability of these innovative approaches to enhance the comprehensive evaluation of ovarian reserve and function.

However, accurately assessing ovarian cellular senescence, which is a key driver of ovarian aging, remains a significant challenge. It is clear that no solitary marker can definitively distinguish between senescence and other states characterized by growth arrest. For assessing the effectiveness of ovarian cellular senescence, employing non-invasive imaging modalities would be optimal. One potential approach could involve using a radioactive β-gal PET tracer for this aim, albeit this is currently in the preliminary stages of development. The non-invasive identification of senescent cells is predicted to be invaluable in forthcoming clinical trials involving pro-senescence and senolytic combinations.

Another challenge is the lack of universally effective senolytic drugs, which were initially developed for general senescence and tested on primary cells. Their effectiveness varies across cell types, making ovarian-specific targeting challenging. CRISPR-Cas9 screening could enable genome-wide identification of new senolytic targets and universal vulnerabilities in senescent ovarian cells. While animal studies have explored senotherapies for ovarian longevity, clinical trials are needed to validate their effectiveness against age-related infertility. Since infertility is non-life-threatening, senotherapy side effects must be minimal to warrant human use.
